# Serological evidence of concurrent Lassa virus and SARS-CoV-2 exposure in Ghana- a cross-sectional study

**DOI:** 10.1186/s12879-025-12385-1

**Published:** 2025-12-20

**Authors:** Elizabeth Obeng-Aboagye, Angelica Daakyire, Portia Owusua Aniapam, Amanda Lamptey, Grace Opoku Gyamfi, Emmanuel Frimpong Gyekye, Christopher Dorcoo, Elvis Suatey Lomotey, Irene Owusu Donkor

**Affiliations:** 1 Department of Epidemiology, Noguchi Memorial Institute for Medical Research, University of Ghana, P.O. Box LG 581, Legon, Accra Ghana; 2 Department of Parasitology, Noguchi Memorial Institute for Medical Research, University of Ghana, P.O.Box LG 581, Legon, Accra Ghana

**Keywords:** SARS-CoV-2, Lassa virus, Dual exposure, Household transmission, Seroprevalence

## Abstract

**Background:**

The COVID-19 pandemic has exposed vulnerabilities in infectious disease surveillance, especially in West Africa where endemic viruses including Lassa fever persist. The overlapping clinical symptoms of these two infections create diagnostic challenges and the possibility of undetected co-infections.

**Methods:**

A retrospective cross-sectional study was conducted using archived serum samples from a nationwide SARS-CoV-2 seroprevalence survey in Ghana. 434 samples across six regions were tested for SARS-CoV-2 total antibodies (IgG/IgM) using the WANTAI ELISA kit and Lassa virus IgG using ReLASV Pan-Lassa-NP-IgG ELISA.

**Results:**

SARS-CoV-2 antibody prevalence was 64.29% (*n* = 279) and Lassa virus IgG prevalence was 20.28% (*n* = 88). Of the cohort of subjects who were seropositive for SARS-CoV-2, 20.79% were also seropositive for LASV IgG. Multivariate analysis revealed household size as a strong risk factor of dual exposure. Individuals from medium-sized households (4–6 persons) (aOR = 8.78, 95% CI: 1.18–65.56, *p* = 0.034) and large households (≥ 7 persons) (aOR = 12.90, 95% CI: 1.99–83.40, *p* = 0.007) had significantly increased odds of dual seropositivity compared to small households. Regional variations were observed, with Greater Accra showing significantly lower odds of dual seropositivity (aOR = 0.13, 95% CI: 0.03–0.51, *p* = 0.004) compared to Ashanti Region.

**Conclusion:**

This study provides serological evidence of SARS-CoV-2 and Lassa virus concurrent exposure in Ghana during the COVID-19 pandemic. This finding suggests large household size as a key driver of dual viral exposure and calls for integrated surveillance systems and targeted interventions in large household settings to reduce concurrent transmission of viruses with pandemic potential.

**Supplementary Information:**

The online version contains supplementary material available at 10.1186/s12879-025-12385-1.

## Introduction

The emergence and rapid global spread of Severe Acute Respiratory Syndrome Coronavirus 2 (SARS-CoV-2), responsible for the COVID-19 pandemic, exposed the vulnerabilities in existing healthcare surveillance systems, particularly in low-and-middle income countries [1, 2]. As of May 2025, a report by the WHO documented over 772 million confirmed COVID-19 cases and more than 7 million associated deaths worldwide [3]. While attention has been largely focused on the COVID-19 pandemic, endemic infectious diseases such as Lassa fever linger and continue to exert a substantial burden on healthcare in West Africa. Lassa fever, an acute viral hemorrhagic illness endemic to West Africa, is estimated to affect between 300,000 and 500,000 individuals annually, resulting in approximately 10,000 deaths [4]. Transmission occurs primarily through exposure to excreta from infected *Mastomys* rodents or through human-to-human contact [5]. Nigeria, a recognized hotspot for Lassa fever, has reported approximately 1620 laboratory confirmed cases and 303 associated deaths between 2024 and 2025 [6]. In this context, West Africa represents a unique epidemiological landscape where the intersection of SARS-CoV-2 and Lassa virus infections presents significant diagnostic, clinical and public health challenges. Although Ghana is not traditionally classified as a high-burden Lassa fever country, sporadic cases and serological evidence of Lassa virus circulation have been documented, warranting increased surveillance and investigation [7]. The clinical presentation of Lassa fever shares considerable overlap with COVID-19, including fever, headache, muscle pain, and respiratory symptoms, creating significant diagnostic challenges in resource-limited settings [8, 9]. This similarity in clinical manifestations increases the risk of misdiagnosis and delays appropriate treatment, potentially contributing to increased morbidity and mortality. The risk of dual exposure may be potentially elevated in large household settings, where increased interpersonal contact and shared living spaces can facilitate transmission of both airborne and contact-transmitted pathogens [10, 11]. While household size and overcrowding (defined as high occupant density per room or living space) are related, they represent distinct risk factors that warrant separate investigation. Furthermore, the concurrent circulation of both pathogens raises concerns about potential dual exposure and co-infections, which may complicate clinical management and epidemiological surveillance efforts [12, 13].

The concept of concurrent viral infections has gained attention in recent years, with studies highlighting the complex interplay between Lassa fever and COVID-19, particularly in Sub-Saharan Africa where these pathogens co-circulate. Research has shown that simultaneous infections may not only complicate clinical diagnosis but also exacerbate disease severity, prolong hospitalization, and increase case fatality rates, particularly in immunocompromised individuals [12, 14]. These findings highlight the need for integrated genomic surveillance systems within existing public health infrastructure to monitor and track the evolution and co-circulation of both emerging and re-emerging viral pathogens and to enhance diagnostic capacity through multiplex panels.

Despite the increasing relevance of dual viral exposure in endemic regions, data on concurrent Lassa fever and COVID-19 exposure and infections remain sparse. A handful of case reports from countries such as Nigeria and Guinea have documented simultaneous infections, primarily among hospitalized patients and healthcare workers [12, 15]. In addition, ecological and modeling studies have begun to explore the broader syndemic implications of overlapping outbreaks on surveillance, healthcare delivery, and disease burden [16]. However, there is a notable absence of large-scale epidemiological studies assessing the prevalence, clinical outcomes, and transmission dynamics of concurrent COVID-19 and Lassa fever exposure. This persistent data gap suggests the urgent need for integrated surveillance efforts and prospective cohort studies to inform evidence-based public health responses in co-endemic settings. This study leveraged archived serum samples from Ghana to retrospectively investigate the seroprevalence and potential dual serological exposure to SARS-CoV-2 and Lassa virus during the COVID-19 pandemic.

## Methods

### Study design

This retrospective study analyzed archived serum samples originally collected during a nationwide seroprevalence survey conducted in Ghana amid the COVID-19 pandemic [17]. The survey instrument and methodology used for the original data collection have been previously published and are described in detail by Owusu Donkor et al. (2023) [17]. The samples were obtained from selected households within Enumeration Areas (EAs) based on the National Sampling Frame provided by the Ghana Statistical Service [18]. As part of the original survey, sociodemographic data, vaccination history, among other health information were collected from all consenting participants. Demographic and health information were collected through structured, interviewer-administered questionnaires in the local language or English, depending on participant preference. Trained field staff conducted face-to-face interviews with participants or their guardians (for minors), collecting information on age, sex, household composition, educational level, occupation, locality type (urban/rural), COVID-19 vaccination status, contact history with suspected or confirmed COVID-19 cases, presence of COVID-19-related symptoms, adherence to COVID-19 prevention protocols, and pre-existing medical conditions. All data were recorded on standardized data collection forms and subsequently entered into electronic databases with quality control checks for completeness and consistency. Of the 6,000 serum samples collected nationwide, 434 samples from six regions (Ashanti, Greater Accra, Eastern, Western, Central, and Brong Ahafo) where preliminary serological screening suggested possible Lassa virus exposure [7] were included in the analysis. All available samples from these regions with sufficient volume for both SARS-CoV-2 and Lassa virus antibody testing were included.

### Laboratory analysis

#### Detection of SARS-CoV-2 antibodies

The WANTAI SARS-CoV-2-Ab sandwich ELISA kit (ref. WS-1096, Beijing WANTAI Biological Pharmacy Enterprise Co., Ltd., Beijing, China) was used to detect total SARS-CoV-2 antibodies (IgG/IgM) in each processed serum sample. This assay detects antibodies against the receptor-binding domain (RBD) of SARS-CoV-2 spike proteins, indicating prior infection, recent infection, or vaccination. Serum samples were processed according to the manufacturer’s protocol using plates pre-coated with recombinant SARS-CoV-2 RBD proteins. Following antibody binding, HRP-conjugated recombinant SARS-CoV-2 antigen was added to form immunocomplexes. Quality controls included manufacturer-supplied negative calibrators (non-reactive newborn calf serum) and positive calibrators (monoclonal anti-RBD antibodies in newborn calf serum). Assay validity required: blank well absorbance < 0.080 at 450 nm, negative calibrator absorbance ≤ 0.100 at 450 nm, and positive calibrator absorbance ≥ 0.190 at 450 nm. Absorbance was measured using a BioTek^®^ Microplate Reader (Gen 5 3.10, BioTek Instruments, Inc., Winooski, VT, USA). Results were calculated by subtracting blank values from test samples and controls, then determining cut-off values by adding 0.16 to the mean negative control absorbance. Sample-to-cut-off ratios > 1 were considered seropositive for SARS-CoV-2 antibodies.

It is important to note that this assay detects total SARS-CoV-2 antibodies (combined IgG/IgM), whereas only IgG antibodies were measured for Lassa virus. This difference in antibody classes detected represents a study limitation, as it does not allow for direct comparison of IgG-specific responses between the two viruses. However, given that the samples were collected during a period when most SARS-CoV-2 infections would have progressed beyond the acute phase, the majority of detected SARS-CoV-2 antibodies likely represent IgG rather than IgM. Nevertheless, we acknowledge that this methodological difference limits precise comparison of seroprevalence rates between the two pathogens.

#### Detection of Lassa virus antibodies

LASV antibody detection was conducted using the ReLASV Pan-Lassa-NP-IgG ELISA kit (RUO-10004, Zalgen Labs, Frederick, Maryland, USA). This kit is designed to detect antibodies against all known Lassa virus lineages (Lineage I-VII), providing broad coverage for serological detection regardless of the specific lineage circulating in Ghana. This pan-lineage detection capability is particularly important given the genetic diversity of LASV across West Africa. All samples were run in duplicates including controls. A threefold serial dilution was done on the standards provided in the kits. The assay involved a two-step procedure using pre-coated LASV NP antigen to capture specific LASV antibodies in the serum samples. A second recombinant antigen conjugated to the enzyme Horseradish peroxidase (HRP-Conjugate) was added to the immunocomplex. The absorbance of detectable antibodies captured in the complete immunocomplex was measured with a BioTek^®^ 149 Microplate Reader (Gen 5 3.10). The limit of detection (LOD) was calculated for each plate using the formula: LOD = Mean + 10 × SD of negative control OD values. A sample was considered seropositive if its concentration exceeded the LOD.

### Data analysis

Data were cleaned and prepared in Microsoft Excel, addressing missing values, duplicates, and inconsistencies before importing into Stata version 17 for statistical analysis. Household size categories were defined based on epidemiological considerations for transmission analysis, with cut-off points chosen to reflect meaningful differences in household crowding levels. The classification was informed by Ghana’s national household patterns as documented in the 2021 Population and Housing Census [18], with categories designed to capture variation in household size that could influence viral transmission dynamics: ‘small’ households (1–3 persons), ‘medium’ households (4–6 persons), and ‘large’ households (≥ 7 persons). It is important to note that household size, as measured in this study, reflects the number of residents rather than household density or overcrowding. While larger households may correlate with crowding conditions, our study did not collect data on number of rooms, floor area, or other housing quality indicators that would allow direct assessment of overcrowding. Therefore, our findings relate specifically to household size as a proxy for potential transmission dynamics. Descriptive statistics, including frequencies and percentages, were used to summarize sociodemographic and clinical characteristics. Bivariate analyses, including cross-tabulations and logistic regression, assessed associations between serostatus for anti-SARS-CoV-2 antibodies and LASV-IgG and sample characteristics. Both outcome variables were binary and analyzed using logistic regression models. Regional differences in sample size were accounted for through logistic regression with region as a categorical variable, which approximately adjusts for unequal group sizes by comparing odds ratios based on proportions rather than absolute numbers. Multicollinearity was assessed using variance inflation factors (mean VIF = 2.76), with all values below 5, indicating no multicollinearity concerns. Model fit was evaluated using the Hosmer-Lemeshow test. Statistical significance was set at *p* ≤ 0.05. Spatial analysis was performed using ArcMap version 10.8 to visualize the geographic distribution of individuals with dual seropositivity based on GPS coordinates, identifying regional patterns and potential hotspots.

## Results

### Background characteristics of study participants

The study included 434 participants with diverse demographic and clinical characteristics. Majority were female (56.91%), and the most represented age group was individuals under 20 years (36.41%), followed by those aged 20–39 years (35.02%). Participants were drawn from six regions across Ghana, with the highest representation from the Ashanti Region (37.10%). Most participants resided in urban areas (62.21%) and belonged to large households with ≥ 7 members (69.82%). Secondary education was most common (42.86%), followed by primary education (35.02%). Only 4.84% had tertiary education. Majority (67.74%) of participants were employed or self-employed. COVID-19 vaccination coverage was low (3%), and most participants (96.31%) had no known pre-existing medical conditions. While 64.98% reported no COVID-19 symptoms, 35.02% experienced symptoms irrespective of infection status. Adherence to COVID-19 preventive measures was poor, with 48.83% reporting no adherence and only 5.48% demonstrating high adherence (Table 1).

**Table 1:** Background characteristics of study participants

Characteristics	Frequency (*n* = 434)	Percentage (%)
***Sex***		
Female	247	56.91
Male	187	43.09
**Age categories**		
< 20	158	36.41
20–39	152	35.02
40–59	80	18.43
60+	44	10.14
**Region**		
Ashanti	161	37.1
Brong Ahafo (Ahafo, Bono, Bono East)	19	4.38
Central	21	4.84
Eastern	100	23.04
Greater Accra	73	16.82
Western	60	13.82
**Household size**		
Small (1–3 people)	66	15.21
Medium (4–6 people)	65	14.98
Large (7 + people)	303	69.82
**Locality type**		
Rural	164	37.79
Urban	270	62.21
**Educational level**		
Never Attended School	30	6.91
Less Than Primary	27	6.22
Primary	152	35.02
Secondary	186	42.86
College/University/Polytechnic	21	4.84
Other (Specify)	18	4.15
**Occupation**		
Employed/Self-employed	294	67.74
Unemployed/Retired	140	32.26
**COVID-19 vaccination status**		
No	421	97
Yes	13	3
**Contact with anyone with flu-like symptoms**		
No	387	89.17
Unknown	17	3.92
Yes	30	6.91
**Contact with anyone with suspected or confirmed COVID-19**		
No	384	88.48
Unknown	48	11.06
Yes	2	0.46
**Pre-existing condition**		
No	418	96.31
Yes	16	3.69
**Presence of COVID-19 symptoms**		
No symptoms	282	64.98
With symptoms	152	35.02
**Level of adherence to COVID-19 protocols**		
High	21	5.48
Low	91	23.76
Moderate	84	21.93
No adherence	187	48.83

*COVID-19, coronavirus disease; %, percentage of participants in category

### Seroprevalence of SARS-CoV-2 total antibodies and Lassa virus IgG

Of the 434 participants, SARS-CoV-2 antibody seropositivity was observed in 64.29% (279/434), while Lassa virus IgG antibodies were detected in 20.28% (88/434). Among individuals seropositive for SARS-CoV-2 antibodies, 20.79% (58/279) were also Lassa virus IgG seropositive. Of these dual-seropositive participants, 53.45% (31/58) resided in urban areas and 46.55% (27/58) in rural areas (Table 2).

**Table 2:** Seroprevalence of SARS-Cov-2 total antibodies and Lassa virus IgG

Characteristics	Frequency (*n* = 434)	Percentage (%)
**SARS-CoV-2 Serostatus**		
Negative	155	35.71
Positive	279	64.29
**LASV IgG Serostatus**		
Negative	346	79.72
Positive	88	20.28
	**SARS-CoV-2 Serostatus**	
**LASV IgG Serostatus**	**Negative (%)**	**Positive (%)**
Negative	125 (80.65)	221 (79.21)
Positive	30 (19.35)	58 (20.79)
**Area of residence of dual seropositive individuals**	(***n*** **= 58**)	
Rural	27	46.55
Urban	31	53.45

*SARS-CoV-2, severe acute respiratory syndrome coronavirus 2; IgG, immunoglobulin G; %, percentage of participants in category

### Associations between background characteristics and dual SARS-CoV-2_Lassa virus seropositivity

Logistic regression analysis was performed to identify factors associated with concurrent seropositivity of SARS-CoV-2 (total antibodies) and Lassa virus (LASV-IgG). In the adjusted model, household size and geographic region were significantly associated with dual seropositivity. Compared to participants from small households (1–3 people), those in medium-sized households (4–6 people) had significantly higher odds of dual seropositivity (aOR = 8.78; 95% CI: 1.18–65.56; *p* = 0.034), while those from large households (≥ 7 people) had the highest odds (aOR = 12.90; 95% CI: 1.99–83.40; *p* = 0.007), suggesting that household crowding influenced dual exposure risk. Participants from the Greater Accra Region had significantly lower odds of dual seropositivity compared to those from the Ashanti Region (aOR = 0.13; 95% CI: 0.03–0.52; *p* = 0.004) (Table 3).

**Table 3:** Logistic regression analysis between dual viral exposure status and participants’ background characteristics

Characteristics	SARS-CoV-2 – LASV IgG		cOR [95% CI]	*p*-value	aOR [95% CI]	*p*-value
	Negative (%)	Positive (%)				
**Sex**						
Female	126 (80.77%)	30 (19.23%)	Ref	.	Ref	.
Male	95 (77.24%)	28 (22.76%)	1.238 [0.693, 2.21]	0.471	1.289 [0.657, 2.531]	0.46
**Age categories**						
20–39	84 (76.36%)	26 (23.64%)	Ref	.	Ref	.
40–59	42 (85.71%)	7 (14.29%)	0.538 [0.216, 1.342]	0.184	0.733 [0.247, 2.169]	0.574
60+	16 (69.57%)	7 (30.43%)	1.413 [0.525, 3.808]	0.494	2.154 [0.525, 8.841]	0.287
< 20	79 (81.44%)	18 (18.56%)	0.736 [0.375, 1.446]	0.374	0.506 [0.221, 1.157]	0.106
**Region**						
Ashanti	69 (75.82%)	22 (24.18%)	Ref	.	Ref	.
Brong Ahafo (Ahafo, Bono, Bono East)	11 (100.00%)	0 (0.00%)	1	.	1	.
Central	10 (83.33%)	2 (16.67%)	0.627 [0.128, 3.083]	0.566	0.356 [0.046, 2.751]	0.322
Eastern	46 (68.66%)	21 (31.34%)	1.432 [0.708, 2.897]	0.318	1.016 [0.446, 2.316]	0.969
Greater Accra	46 (90.20%)	5 (9.80%)	0.341 [0.12, 0.965]	0.043	0.133 [0.034, 0.517]	0.004
Western	39 (82.98%)	8 (17.02%)	0.643 [0.262, 1.581]	0.336	0.733 [0.23, 2.334]	0.599
**Household size**						
small (1–3 persons)	36 (92.31%)	3 (7.69%)	Ref	.	Ref	.
medium (4–6 persons)	32 (82.05%)	7 (17.95%)	2.625 [0.626, 11.012]	0.187	8.783 [1.177, 65.555]	0.034
large (7 + persons)	153 (76.12%)	48 (23.88%)	3.765 [1.11, 12.772]	0.033	12.901 [1.996, 83.402]	0.007
**Area of residence**						
Rural	74 (73.27%)	27 (26.73%)	Ref	.	1	.
Urban	147 (82.58%)	31 (17.42%)	0.578 [0.321, 1.039]	0.067	0.637 [0.31, 1.309]	0.219
**Educational level**						
College/University/Polytechnic	13 (81.25%)	3 (18.75%)	Ref	.	Ref	.
Less Than Primary	14 (87.50%)	2 (12.50%)	0.619 [0.089, 4.316]	0.628	0.997 [0.097, 10.287]	0.998
Never Attended School	14 (82.35%)	3 (17.65%)	0.929 [0.158, 5.448]	0.935	2.181 [0.242, 19.633]	0.487
Other (Specify)	5 (62.50%)	3 (37.50%)	2.6 [0.387, 17.451]	0.325	2.751 [0.293, 25.836]	0.376
Primary	76 (78.35%)	21 (21.65%)	1.197 [0.312, 4.596]	0.793	2.426 [0.427, 13.799]	0.318
Secondary School	99 (79.20%)	26 (20.80%)	1.138 [0.302, 4.293]	0.849	1.996 [0.387, 10.306]	0.409
**Occupation**						
employed/self-employed	151 (79.06%)	40 (20.94%)	Ref	.	Ref	.
unemployed/retired	70 (79.55%)	18 (20.45%)	0.971 [0.52, 1.812]	0.926	1.065 [0.455, 2.492]	0.885
**COVID-19 vaccination status**						
No	212 (79.40%)	55 (20.60%)	Ref	.	Ref	.
Yes	9 (75.00%)	3 (25.00%)	1.285 [0.336, 4.906]	0.714	4.868 [0.699, 33.88]	0.11
**Contact with anyone with flu-like symptoms**						
No	195 (78.95%)	52 (21.05%)	Ref	.	Ref	.
Unknown	13 (100.00%)	0 (0.00%)	1	.	1	.
Yes	13 (68.42%)	6 (31.58%)	1.731 [0.628, 4.774]	0.289	1.74 [0.462, 6.549]	0.413
**Contact with anyone with suspected or confirmed COVID-19**						
No	189 (78.10%)	53 (21.90%)	Ref	.	Ref	.
Unknown	31 (88.57%)	4 (11.43%)	0.46 [0.155, 1.362]	0.161	1.644 [0.375, 7.197]	0.509
Yes	1 ( 50.00%)	1 (50.00%)	3.566 [0.219, 57.972]	0.371	0.601 [0.018, 20.044]	0.776
**Pre-existing condition**						
No	211 (79.03%)	56 (20.97%)	Ref	.	Ref	.
Yes	10 (83.33%)	2 (16.67%)	0.754 [0.161, 3.538]	0.72	1.086 [0.129, 9.154]	0.939
**Level of adherence to COVID-19 protocols**						
High	15 (83.33%)	3 (16.67%)	Ref	.	Ref	.
Low	46 (79.31%)	12 (20.69%)	1.304 [0.324, 5.252]	0.709	1.055 [0.183, 6.063]	0.953
Moderate	52 (80.00%)	13 (20.00%)	1.25 [0.314, 4.971]	0.751	1.211 [0.227, 6.471]	0.823
No adherence	96 (76.80%)	29 (23.20%)	1.51 [0.409, 5.583]	0.536	1.834 [0.357, 9.413]	0.467

*aOR, adjusted odds ratio; CI, confidence interval; SARS-CoV-2, severe acute respiratory syndrome coronavirus 2; IgG, immunoglobulin G

### Spatial distribution of dual seropositivity

**Fig. 1:**
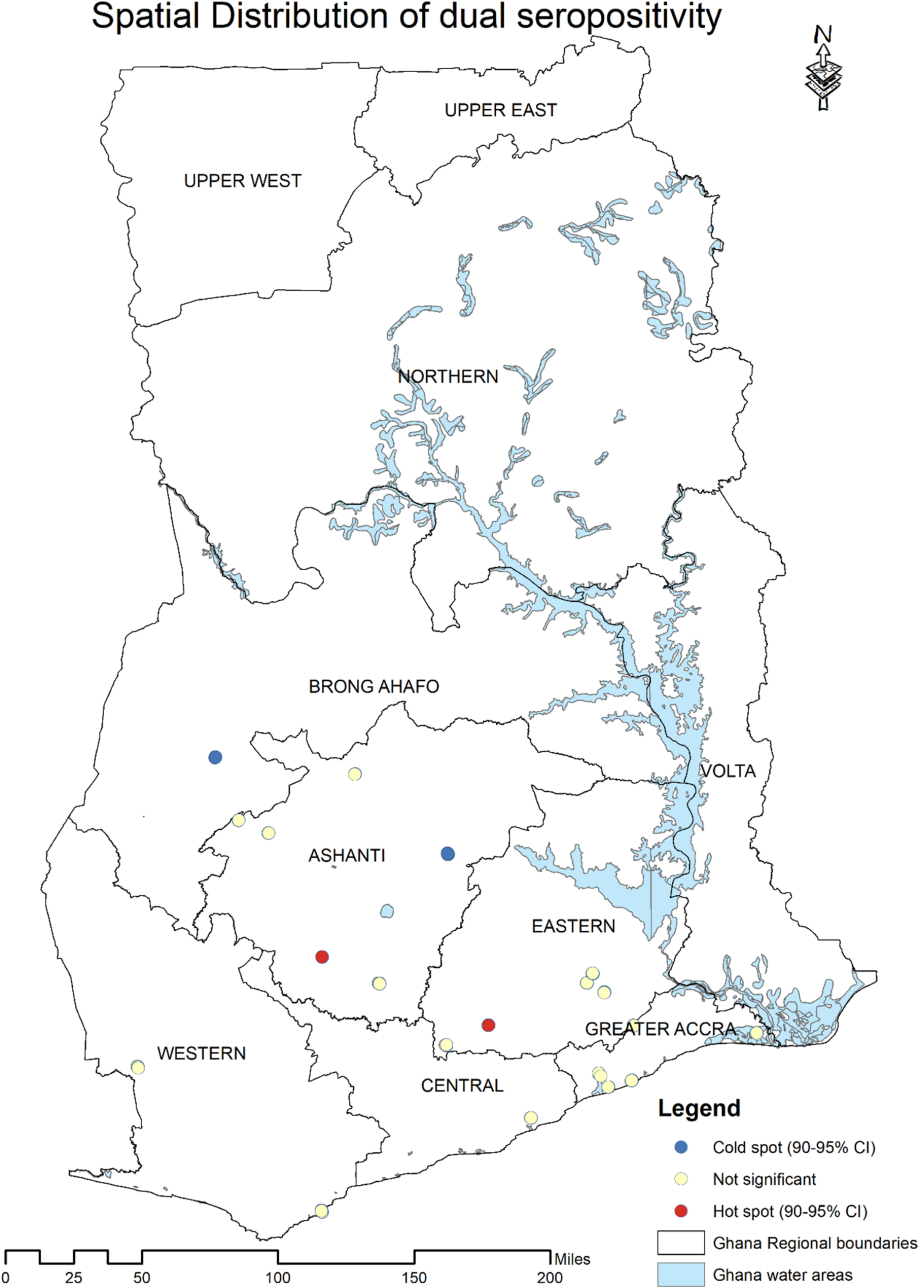
Spatial distribution of dual SARS-CoV-2 and Lassa virus seropositivity across six regions in Ghana. Map shows geographic distribution of 58 individuals with concurrent seropositivity. Moran’s I analysis revealed no significant spatial clustering (I = 0.024, Z-score = 0.805, *p* = 0.421), indicating dispersed rather than clustered distribution. Created using ArcMap version 10.8

A hotspot analysis was performed using the Getis-Ord Gi* statistics to identify geographic locations with significantly high (hot spots) or low (cold spots) concentrations of dual seropositive individuals. To analyze the clustering pattern of dual seropositivity cases, a spatial autocorrelation analysis was performed with Moran’s I. The results showed a Moran’s I value slightly above zero (0.024), indicating minimal clustering, with a Z-score (0.805) and p-value (0.421). This indicates no statistically significant spatial clustering in the data, suggesting dual seropositivity is dispersed over space rather than concentrated in specific geographic areas (Fig. 1).

## Discussion

This study provides serological evidence of concurrent SARS-CoV-2 and Lassa virus exposure in Ghana, revealing a dual seroprevalence rate of 20.79% among COVID-19 positive individuals and 13.36% in the overall study population. These findings represent systematic data on SARS-CoV-2 and Lassa virus concurrent exposure from West Africa, contributing important epidemiological evidence to the limited literature on this topic. The observed concurrent exposure rate of 20.79% among individuals who tested positive for SARS-CoV-2 is notably higher than the expected rate of approximately 13.04%, calculated (using the multiplicative rule of probability) under the assumption of independent exposure to each virus. This discrepancy suggests a potential non-random association between the two infections. Several factors may contribute to this elevated dual seroprevalence, including shared risk environments such as densely populated households, inadequate sanitation, and restricted access to healthcare services. These factors facilitate the spread of both respiratory and zoonotic infections in endemic regions [19, 20], highlighting a notable overlap between two significant viral threats in Ghana. This finding aligns with emerging evidence from other parts of West Africa where co-infections with COVID-19 and endemic viruses have been reported [21]. While our study utilized a pan-Lassa virus assay capable of detecting antibodies against all known LASV lineages, we did not perform viral genomic characterization to determine which specific lineage(s) are circulating in Ghana. Previous studies have identified lineage II LASV in neighboring West African countries [22, 23], though comprehensive genomic surveillance data for Ghana remain limited. Future studies incorporating genomic sequencing would provide valuable insights into the molecular epidemiology of LASV in Ghana and help inform regionally tailored prevention strategies.

Notably, the most striking result from our analysis was the strong association between household size and the risk of dual viral exposure. Participants from medium-sized households (4–6 persons) showed nearly 9-fold increased odds of concurrent viral exposure (aOR = 8.78, 95% CI: 1.18–65.56, *P* = 0.034), while those from large households (≥ 7 persons) demonstrated nearly 13-fold increased odds (aOR = 12.90, 95% CI: 1.99–83.40, *p* = 0.007) compared to small households. This finding aligns with established literature on how large household sizes potentially facilitate the transmission of respiratory viruses and contact-transmitted pathogens [10, 11]. While we measured household size (number of residents) rather than household density (people per room or per square meter), the observed association likely reflects several interrelated factors. Larger households typically involve increased interpersonal interaction and shared indoor areas with possibly insufficient ventilation, which may contribute to the airborne spread of SARS-CoV-2 [11]. Additionally, larger household size may correlate with more limited living space per person, potentially increasing the likelihood of contact with contaminated surfaces or vectors, thereby potentially facilitating Lassa virus transmission through infected rodent excreta or contaminated household items. However, it is important to acknowledge that without direct measures of housing quality, room density, or square footage, we cannot definitively attribute this association to overcrowding per se. Future studies incorporating objective measures of household density (such as people-per-room ratios) would help distinguish whether the increased risk is driven primarily by the number of household members, the physical density of living arrangements, or both factors in combination.

Interestingly, residing in Greater Accra was associated with lower odds of concurrent viral exposure (aOR = 0.13, 95% CI: 0.03–0.51, *p* = 0.004) compared to Ashanti Region, suggesting important regional differences in exposure risk, healthcare access, or population susceptibility. This geographic variation may reflect several factors including differences in healthcare infrastructure, population density, environmental conditions, and cultural practices that influence exposure to both pathogens. Greater Accra, as Ghana’s capital region, may have better healthcare infrastructure and disease surveillance systems, potentially leading to earlier detection and isolation of cases, thereby reducing transmission opportunities. Additionally, urban environments in Greater Accra may have different housing patterns and sanitation infrastructure compared to other regions, potentially affecting exposure to Lassa virus vectors. However, this does not eliminate the risk of dual viral exposure in urban areas, as over half of the individuals with dual viral exposure were from urban settings. This underscores the complex interplay of environmental, behavioral, and socioeconomic factors in disease dynamics. The regional differences observed in our study highlight the need for tailored public health approaches that consider local epidemiological contexts. Understanding these geographic variations is crucial for resource allocation and the development of region-specific prevention strategies.

Our findings have important implications for clinical practice and diagnostic approaches in Ghana and similar West African settings. Given the substantial dual exposure rate observed (20.79% among SARS-CoV-2 seropositive individuals) and the overlapping clinical presentations of Lassa fever and COVID-19, we recommend enhanced diagnostic vigilance in specific contexts. Healthcare providers in regions with documented Lassa virus circulation (particularly Ashanti, Eastern, and Western regions based on our findings) should maintain a high index of suspicion for possible Lassa fever in patients presenting with severe febrile illness, especially during respiratory viral outbreaks.

While universal LASV testing for all serious viral infections may not be feasible given resource constraints, we advocate for a risk-stratified approach to LASV testing that considers: (1) geographic location (regions with documented LASV seropositivity) (2), household characteristics (large households based on our risk factor analysis) (3), clinical presentation (severe febrile illness with hemorrhagic manifestations or failure to improve with standard treatment), and (4) epidemiological context (concurrent circulation of respiratory viruses). Implementation of multiplex diagnostic platforms capable of simultaneously detecting SARS-CoV-2, Lassa virus, and other endemic pathogens would be particularly valuable in high-risk settings.

Furthermore, our findings support the need for integrated disease surveillance systems that can detect co-circulation of multiple viral threats rather than vertical, disease-specific surveillance programs. Such integrated approaches would improve early detection of dual exposures, guide appropriate clinical management, and inform public health response strategies during outbreaks.

## Conclusion

This study provides important serological evidence on concurrent exposure to SARS-CoV-2 and Lassa virus in Ghana, revealing substantial dual exposure rates and identifying large household size as a major risk factor which can increase co-infection rate via multiple introductions within a short time, making control measures like isolation harder to implement. While we measured household size rather than overcrowding specifically, the strong association suggests that household-level factors merit further investigation. Regional variations in dual viral exposure risk suggest the need for locally adapted surveillance and prevention strategies. These findings have significant implications for public health policy, clinical practice, and disease surveillance in West Africa. Enhanced surveillance systems and region-specific prevention strategies should be considered to address the dual burden of these important pathogens. Further research is needed to understand the clinical outcomes, immunological interactions, and optimal management strategies for patients with dual viral exposure.

### Limitations

Several limitations should be considered when interpreting our findings. First, we measured household size rather than household crowding. Collecting detailed housing information including number of sleeping rooms, square footage, and people-per-room ratios would better characterize the independent effects of household size versus household density on dual viral exposure risk. Second, the cross-sectional design limits our ability to establish temporal relationships between the two infections or determine causality. Longitudinal studies would be needed to understand the sequence of infections and their potential interactions. Third, the serological approach cannot definitively establish whether the dual viral exposure occurred simultaneously or sequentially, which has important implications for understanding disease burden and clinical management. An additional limitation is the detection of different antibody classes for the two viruses: total antibodies (IgG/IgM) for SARS-CoV-2 versus IgG only for Lassa virus. This methodological difference limits direct comparability of seroprevalence rates and may affect interpretation of concurrent exposure. Lastly, the relatively small number of confirmed cases with dual viral exposure (*n* = 58) limited our statistical power to detect associations with some variables that might be clinically relevant. Larger studies with multi-site recruitment would strengthen the evidence base.

### Recommendations

Future research should focus on prospective longitudinal studies to better understand the dynamics of dual viral exposure and their clinical consequences. Additionally, genomic surveillance approaches could provide valuable insights into the molecular epidemiology of co-circulating pathogens and help distinguish between genuine concurrent exposures and cross-reactive antibody responses. Investigation of the immunological interactions between co-circulating pathogens could also inform vaccine development and treatment strategies. Research into the environmental and behavioral factors, including household density assessment, that facilitate dual viral exposure could inform targeted surveillance and prevention strategies.

## Supplementary Information

Below is the link to the electronic supplementary material.

Supplementary Material 1

## Data Availability

The datasets used and/or analyzed during the current study are available from the corresponding author on reasonable request. Due to privacy and ethical considerations, raw individual-level data cannot be shared publicly. Aggregated data supporting the conclusions of this article are included within the article. This study followed the STROBE guidelines for reporting cross-sectional studies, and a completed STROBE checklist is provided as Supplementary File 1.

## Abbreviations

CI: Confidence Interval
COVID-19: Coronavirus Disease 2019
ELISA: Enzyme-Linked Immunosorbent Assay
IgG: Immunoglobulin G
IgM: Immunoglobulin M
LOD: Limit of Detection
OR: Odds Ratio
QC: Quality Control
RT-PCR: Reverse Transcription Polymerase Chain Reaction
SARS-CoV-2: Severe Acute Respiratory Syndrome Coronavirus 2
STROBE: Strengthening the Reporting of Observational Studies in Epidemiology
WHO: World Health Organization
