# Supplementary material for: Serological evidence of concurrent Lassa virus and SARS-CoV-2 exposure in Ghana- a cross-sectional study
Source: BMC Infect Dis. 2025 Dec 20;26:132. doi: 10.1186/s12879-025-12385-1 (PMC12831435; doi:10.1186/s12879-025-12385-1)
Supplement: Supplementary file 1 — Supplementary Material 1 [file 12879_2025_12385_MOESM1_ESM.docx]

**STROBE Checklist**

**Title and abstract**

The title identifies the study as a cross-sectional analysis. The abstract is structured (Background, Methods, Results, Conclusion) and summarizes study design, methods, and findings.

**Background/rationale**

The introduction explains the co-circulation of SARS-CoV-2 and Lassa virus in West Africa and the rationale for assessing concurrent serological exposure.

**Objectives**

The study aims to estimate concurrent seroprevalence of SARS-CoV-2 and Lassa virus and to assess demographic and household-level risk factors.

**Study design**

Cross-sectional serological analysis of archived samples from a nationwide survey.

**Setting**

Nationwide survey in Ghana across six administrative regions. Serum samples collected February-December 2021.

**Participants**

434 serum samples randomly selected from approximately 6,000 participants of a nationwide survey; eligibility based on availability of stored serum and consent.

**Variables**

Outcomes: SARS-CoV-2 IgM/IgG, Lassa virus IgG. Exposures: demographics, household characteristics. Covariates: age, sex, region, household size.

**Data sources/measurement**

Serology performed using WANTAI SARS-CoV-2 ELISA and ReLASV ELISA kits. Cut-offs defined by LOD formula. Demographic/household data from survey records.

**Bias**

Selection bias reduced by random sampling across six regions. Laboratory bias minimized by duplicate testing and QC controls.

**Study size**

Sample size (n=434) chosen based on resource constraints and to ensure regional representation. No formal power calculation was performed.

**Quantitative variables**

Age treated as both continuous and categorical. Household size grouped into small (≤5) vs large (>5).

**Statistical methods**

Seroprevalence calculated with 95% CI. Logistic regression for associations. P<0.05 considered significant. Analyses performed in Stata

**Results- Participants**

Flow described: 6,000 surveyed: 434 samples randomly selected and tested. Numbers reported in text and Table 1.

**Descriptive data**

Table 1 shows demographics (age, sex, region, household size) of participants.

**Outcome data**

Tables and text report numbers and proportions positive for SARS-CoV-2 IgM/IgG, Lassa IgG, and dual seropositivity.

**Main results**

Logistic regression results presented with ORs and 95% CI. Adjusted for age, sex, region, and household size.

**Other analyses**

Hotspot analysis using ArcMap; subgroup analysis by region and household size.

**Discussion- Key results**

Summarized in relation to objectives: dual seropositivity higher than expected by chance, suggesting non-random association.

**Limitations**

Acknowledges cross-reactivity, selection of subset (n=434), and lack of IgM testing for Lassa due to funding constraints.

**Interpretation**

Findings interpreted cautiously in context of COVID-19 and endemic Lassa fever; relevance for surveillance emphasized.

**Generalizability**

Results generalizable to surveyed Ghanaian regions but limited by sample size and archived subset.

**Funding**

Funding support acknowledged; funders had no role in design, analysis, or manuscript preparation
